# *S100a4* upregulation in *Pik3ca*H1047R;*Trp53*R270H;*MMTV-Cre*-driven mammary tumors promotes metastasis

**DOI:** 10.1186/s13058-019-1238-5

**Published:** 2019-12-27

**Authors:** Wenlin Yuan, Leonard D. Goldstein, Steffen Durinck, Ying-Jiun Chen, Thong T. Nguyen, Noelyn M. Kljavin, Ethan S. Sokol, Eric W. Stawiski, Benjamin Haley, James Ziai, Zora Modrusan, Somasekar Seshagiri

**Affiliations:** 1 Department of Molecular Biology, Genentech Inc, 1 DNA Way, South San Francisco, CA 94080 USA; 2 Department of Bioinformatics and Computational Biology, Genentech Inc, 1 DNA Way, South San Francisco, CA 94080 USA; 3 Department of Cancer Signaling, Genentech Inc, 1 DNA Way, South San Francisco, CA 94080 USA; 4 Foundation Medicine Inc., 150 Second Street, Cambridge, MA 02141 USA; 5 Research and Development Department, MedGenome Inc., Foster City, CA 94404 USA; 6 Department of Pathology, Genentech Inc, 1 DNA Way, South San Francisco, CA 94080 USA; 7 SciGenom Research Foundation, Bangalore, 560099 India

**Keywords:** *Pik3ca*H1047R, *Trp53*R270H, *S100a4*, Mammary tumors, Breast cancer, Metastasis

## Abstract

**Background:**

*PIK3CA* mutations are frequent in human breast cancer. *Pik3ca*H1047R mutant expression in mouse mammary gland promotes tumorigenesis. *TP53* mutations co-occur with *PIK3CA* mutations in human breast cancers. We previously generated a conditionally activatable *Pik3ca*H1047R;*MMTV-Cre* mouse model and found a few malignant sarcomatoid (spindle cell) carcinomas that had acquired spontaneous dominant-negative *Trp53* mutations.

**Methods:**

A *Pik3ca*H1047R;*Trp53*R270H;*MMTV-Cre* double mutant mouse breast cancer model was generated. Tumors were characterized by histology, marker analysis, transcriptional profiling, single-cell RNA-seq, and bioinformatics. Cell lines were developed from mutant tumors and used to identify and confirm genes involved in metastasis.

**Results:**

We found *Pik3ca*H1047R and *Trp53*R270H cooperate in driving oncogenesis in mammary glands leading to a shorter latency than either alone. Double mutant mice develop multiple histologically distinct mammary tumors, including adenocarcinoma and sarcomatoid (spindle cell) carcinoma. We found some tumors to be invasive and a few metastasized to the lung and/or the lymph node. Single-cell RNA-seq analysis of the tumors identified epithelial, stromal, myeloid, and T cell groups. Expression analysis of the metastatic tumors identified *S100a4* as a top candidate gene associated with metastasis. Metastatic tumors contained a much higher percentage of epithelial–mesenchymal transition (EMT)-signature positive and *S100a4*-expressing cells. CRISPR/CAS9-mediated knockout of *S100a4* in a metastatic tumor-derived cell line disrupted its metastatic potential indicating a role for *S100a4* in metastasis.

**Conclusions:**

*Pik3ca*H1047R;*Trp53*R270H;*MMTV-Cre* mouse provides a preclinical model to mimic a subtype of human breast cancers that carry both *PIK3CA* and *TP53* mutations. It also allows for understanding the cooperation between the two mutant genes in tumorigenesis. Our model also provides a system to study metastasis and develop therapeutic strategies for *PIK3CA*/*TP53* double-positive cancers. S100a4 found involved in metastasis in this model can be a potential diagnostic and therapeutic target.

## Background

*PIK3CA* gene mutation or amplification occurs in 26–36% of breast cancers [1, 2]. The most recurrent *PIK3CA* mutation, H1047R, is constitutively active and promotes PI3K signaling [3]. Several mouse transgenic or knock-in models have been developed to study *PIK3CA*-driven oncogenesis [4–10]. Given the importance of PI3K signaling in cancer, several drugs targeting p110α and/or other members of the p110α family have been developed and are in clinical development [11]. Tumor suppressor *TP53* is a commonly mutated cancer gene [2]. In human breast cancer, *TP53* mutations occur in 37–46% of the cases [1, 2], and in about 13% of the cases, *PIK3CA* and *TP53* mutations co-occur [1].

We previously developed a conditionally activatable *Pik3ca*H1047R;*MMTV-Cre* mouse model where we found expression of *Pik3ca*H1047R from the endogenous locus using *MMTV-Cre* primarily led to the development of benign mammary fibroadenomas [7]. In addition to benign tumors, we also found a few malignant sarcomatoid (spindle cell) carcinomas that had acquired spontaneous dominant-negative *Trp53* mutations [7]. Given this and also the co-occurrence of *PIK3CA* and *TP53* mutations in human breast cancers, we developed a *Pik3ca*H1047R;*Trp53*R270H;*MMTV-Cre* double mutant model by crossing the *Pik3ca*H1047R;*MMTV-Cre* mice with *Trp53*R270H flox mice [12]. We found that the *Pik3ca*H1047R;*Trp53*R270H;*MMTV-Cre* double mutant mice had a shorter latency of 36.6 weeks for tumor development compared to 62 weeks in *Pik3ca*H1047R;*MMTV-Cre* mice. Some of the double mutant animals also developed metastasis. Here, we report *S100a4* to be a candidate gene involved in metastasis in this model.

## Methods

### Analysis of *PIK3CA* and *TP53* mutations in human cancers

*PIK3CA* and *TP53* alterations were analyzed in 111,176 patient tumor samples, including 4485 breast cancer samples, sequenced with comprehensive genomic profiling (CGP) [13]. CGP was performed in a Clinical Laboratory Improvement Amendments (CLIA)-certified, CAP (College of American Pathologists)-accredited laboratory (Foundation Medicine, Inc., Cambridge, MA, USA) on all-comers during the course of routine clinical care. Approval was obtained from the Western Institutional Review Board (Protocol No. 20152817). Hybrid capture was performed for all coding exons from 285 to 315 cancer-related genes plus select introns from 28 genes frequently rearranged in cancer. We assessed all classes of genomic alterations (GA) including short variant, copy number, and rearrangement alterations, as previously described [9]. All known or likely pathogenic alterations were included in this analysis.

### Generation of *Pik3ca*H1047R;*Trp53*R270H;*MMTV-Cre* mice

**Fig. 1:**
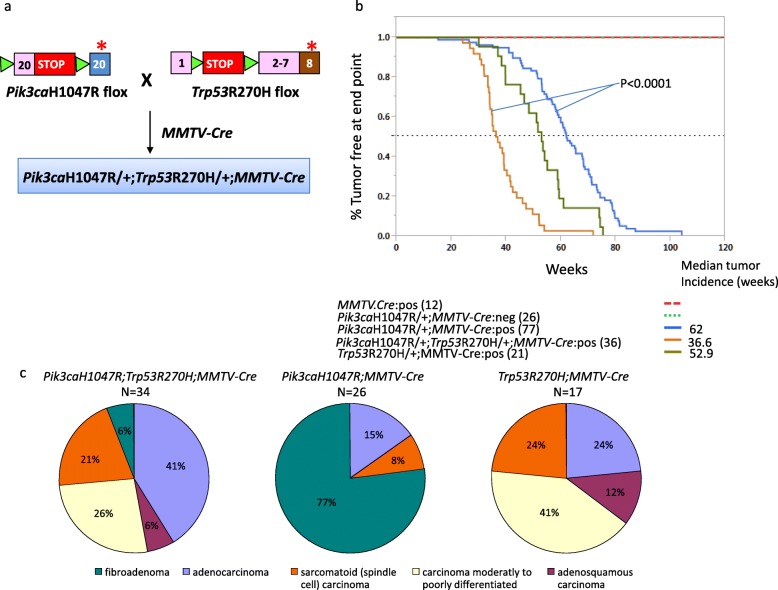
Tumor-free survival of double mutant *Pik3ca*H1047R;*Trp53*R270H;*MMTV-Cre* mice. **a** *Pik3ca*H1047R;*MMTV-Cre* mice were crossed with *Trp53*R270Hflox mice to obtain the *Pik3ca*H1047R;*Trp53*R270H;*MMTV-Cre* mice. **b** Kaplan–Meier plot depicting tumor-free survival of the indicated mouse lines. **c** Pie-chart representation of tumor types observed

*Pik3ca*H1047R; *MMTV-Cre* mice were crossed with *Trp53*R270Hflox mice obtained from Dr. Tyler Jacks, Massachusetts Institute of Technology [12], to generate *Pik3ca*H1047R;*Trp53*R270H;*MMTV-Cre* mice (Fig. 1a). Compound mutant mice were generated on C57BL/6 genetic background. All mice were maintained in our animal facility as per the Institutional Animal Care and Use Committee (IACUC) guidelines. Mice were palpated weekly for the presence of tumors. Mice that showed any body condition score < 2, a hunched posture, and > 20% loss of body weight (together designated as under-conditioned mice) were euthanized as per IACUC guidelines. Our study endpoint included mice with tumors that reached ~ 2500 mm^3^ and the under-conditioned situation mentioned above.

### Histological and immunofluorescence analysis

Five-micrometer, formalin-fixed, paraffin-embedded specimens were used for routine hematoxylin and eosin (H&E) staining and histology evaluation. Immunofluorescence staining was performed using 10-μm sections Tissue-Tek OCT (Sakura Finetek, CA) embedded frozen samples. Sectioned samples were fixed in 4% paraformaldehyde for 10 min and then blocked for 30 min with PBT (PBS with 0.1% Triton) containing 1% BSA. The blocked sections were then stained with appropriate primary antibody diluted in PBT with 0.1% BSA overnight at 4 °C in a humidified chamber. The slides were washed three times in PBT and then incubated with appropriate secondary antibodies for 60 min at room temperature in a humidified chamber. Unbound secondary antibody was removed by washing with PBT. Prolong Gold anti-fade reagent (Thermo Scientific, CA) was used to mount the slides. Primary antibodies used in the study were cytokeratin 5 (ab53121, Abcam, MA), cytokeratin18 (ab668, Abcam, MA), ERα (Ab-21, Thermo Scientific, CA), progesterone receptor (ab2764, Abcam, MA), and vimentin (ab45939, Abcam, MA). Appropriate secondary antibodies conjugated with Alexa 488 or 647 (Molecular probes, OR) were used for detecting the bound primary antibody.

### Development of primary tumor cell lines

Tumor tissue was digested in PBS with 4 mg/ml collagenase (C0130-1G, Sigma, MO) and 5 mg/ml hyaluronidase (H2126-1G, Sigma, MO) for 1 h at 37 °C to generate a cell suspension. Cells were then cultured in Epicult B basal medium (05610, Stemcell Technology, BC, Canada) with MEGM mammary epithelial cell growth medium containing SingleQuots supplements for growth factors (CC-4136, Lonza, MD), 1 ng/ml bFGF (MilliporeSigma, MA), and heparin (1:100 dilution; 07980, Stemcell Technology, BC, Canada).

### Gene expression analysis

Microarray data was collected as previously described [7]. Data normalization and differential gene expression analysis of the Agilent microarray data was performed using limma [14]. Metastatic sarcomatoid carcinoma group used in the expression analysis consisted of a *Pik3ca*H1047R;*Trp53*R270H;*MMTV-Cre* primary tumor (tumor 1), a lung metastatic tumor (tumor 2) derived by tail vein injection involving a cell line derived from tumor 1, a *Pik3ca*H1047R;*MMTV-Cre* primary tumor (tumor 3) with a spontaneous *Trp53*R245H mutation previously described [7], a lung metastatic tumor (tumor 4) derived by tail vein injection involving a cell line derived from tumor 3, and a *Trp53*R270H;*MMTV-Cre* primary tumors (tumor 5). Five adenocarcinomas *Pik3ca*H1047R;*Trp53*R270H;*MMTV-Cre* primary tumors were also used in the expression analysis. Cell lines developed from the adenocarcinoma were not metastatic in the tail vein injection model.

RNA-seq libraries were prepared using the TruSeq RNA Sample Preparation kit (Illumina, CA). The libraries were multiplexed three per lane and sequenced on the HiSeq platform to obtain, on average, ~ 68 million paired-end (2 × 75-bp) reads per sample. All sequencing reads were evaluated for quality using the Bioconductor ShortRead package.

### Single-cell RNA-seq

Single-cell suspension from the tumors was obtained using gentleMACS™ Dissociator (Milteny, Germany) and the associated tumor dissociation protocol. Single-cell RNA-seq data were generated using 10x Genomics Chromium Single Cell 3′ kits (v1 & v2). The cell density and viability of single-cell suspension was determined using a Vi-CELL (Beckman Coulter, CA) cell counter. The cDNA and libraries were prepared following the manufacturer’s (10x genomics, CA) instructions. Each library was quantified (Bioanalyzer High Sensitivity DNA kit (Agilent, CA) and Kapa Library quantification (Kapa Biosystems, MA) kit and sequenced on HiSeq2500/4000 (Illumina, CA).

### Single-cell RNA-seq data processing and analysis

Single-cell RNA-seq data were processed with a custom pipeline. Briefly, reads were demultiplexed based on perfect matches to expected cell barcodes. Transcript reads were aligned to the mouse reference genome (GRCm38) using GSNAP (2013-10-10) only considering uniquely mapped reads [15]. Per-gene transcript counts were determined based on the number of unique UMIs for mapped reads overlapping exons in sense orientation, allowing for one mismatch when collapsing UMI sequences. To be considered for downstream analysis, cells were required to exceed a minimum number of detected transcripts, where a sample-specific cutoff was set to 0.1 times the total transcript count for cells at rank 30 (the 99th percentile for 3000 cells). Per-gene transcript counts were normalized by dividing counts for each cell by a cell-specific scale factor, calculated as the total transcript count for a given cell, divided by the median total transcript count across cells. Normalized counts were then transformed using a log2(x + 1) transformation. PCA was performed on the 1000 most variable genes. The top 20 principal components were used for model-based clustering with the R software package mclust (5.3) [16]. The final mixture model (VEV) and number of clusters (*n* = 19) were chosen based on minimal BIC score when considering up to 20 clusters. We identified genes enriched for each cluster using a non-parametric Wilcoxon rank-sum test. After inspecting enriched genes, clusters were merged into superclusters corresponding to epithelial cells (*n* = 2313), stromal cells (*n* = 369), T cells (*n* = 638), monocytes/macrophages (*n* = 1423), and putative cell multiplets (*n* = 252). Cell multiplets were not included in further analysis. Cells were also analyzed for expression of EMT genes (*Snai1*, *Twist1*, *Zeb1*, *Zeb2*) and *S100a4*.

### *S100a4* CRISPR/Cas9 knockout cell lines

A lentiviral sgRNA (guide RNA) and Cas9 expression vector, pLKO_SHC201_AIO_CMV_Cas9Puro, was constructed by gene synthesis (Genscript Inc., NJ) followed by cloning into the pLKO.5 backbone (Sigma-Aldrich, MO). sgRNAs targeting mouse *S100a4* were identified as previously described [17]. The sgRNA targeting sequences are S100a4-2: 5′- gctcaaggagctactgacc-3′ (exon2) and S100a4-3: 5′-gacaatgaagttgacttcc-3′ (exon3). sgRNAs were individually synthesized and cloned into pLKO_SHC201_AIO_CMV_Cas9Puro (Genscript Inc., NJ).

To generate lentivirus, low-passage HEK-293 cells were transiently transfected with a combination of pLKO_SHC201_AIO_CMV_Cas9PURO_flox_gRNA expression plasmid, Δ8.9 packaging plasmid, and VSVG envelop plasmid using Lipofectamine 2000 (Life Technologies, CA) in Opti-MEM I media (Life Technologies, CA). After 72 h, lentiviruses were concentrated from cultured supernatants with Lenti-X Concentrator (Clontech, CA). Cell lines were infected with lentivirus and 8 μg/ml polybrene by two rounds of spin infection and selected on 2 μg/ml puromycin. Gene knockout was confirmed by aligning RNA-seq reads to the mouse genome (GRCm38) using GSNAP [15]. Allele frequencies of indels were computed by counting every indel event in reads aligning to the on-target position.

### Cell proliferation assay

For each knockout line, ~ 5000 cells in 12 replicates were plated in a 384-well plate (Corning 3595, CA) coated with Collagen I. Cells were grown at 37 °C in the presence of 5% CO_2_ and imaged at × 10 magnification using an IncuCyte Zoom Live-content imaging system (Essen Bioscience, MI). Images were acquired every 2 h for 4 days, with four images per well. Data was analyzed using IncuCyte analysis software to detect and quantify live cell confluence (phase-contrast). Averages with standard error of the mean at each time point were plotted.

### Experimental metastasis assay

Tumor-derived cell cultures were harvested at 70% confluency, counted, and dispersed in Hanks Balanced Buffer Solution (HBSS) at a concentration of 1 million cell per microliter. Target mouse was held with a mouse restrainer, with its tail sticking out of the small opening in the back of the restrainer. About 200,000 cells in a 200 μl were injected into the mouse tail vein using a 1-ml syringe. For the initial metastatic potential using tail vein injection, we analyzed three adenocarcinoma and three sarcomatoid carcinoma cell lines. We used three mice per line (a total of 18 mice) for this study. Mice were monitored for weight loss and labored breathing for 3 months. Lung tissue was harvested at the end of the study or when animals were euthanized under the study protocol guidelines. Gross lung examination was performed for the presence of metastasis, and metastasis was confirmed further by histological analysis. Metastasis studies were performed using the CRISPR knockout lines in a similar manner.

## Results

### Co-occurrence of *PIK3CA* and *TP53* mutations

We observed spontaneous *Trp53* mutations in a *Pik3ca*-mutant-driven mouse model of breast cancer that we had previously developed [7]. In an effort to understand this further, we assessed the co-occurrence of *PIK3CA* and *TP53* in human cancers in a dataset of 111,176 patients involving 56 major cancer types [13]. Overall *TP53* was mutated in 53% of the samples and *PIK3CA* was mutated in 13% of the samples. In primary breast cancers, 62% (2763/4485) of the samples carried a *TP53* mutation, making it the most frequently mutated gene. This was followed by *PIK3CA* which was mutated in 31% (1389/4485) of the breast cancer samples (Additional file 1: Figure S1). Overall, we found 15% (678/4485) of the breast cancers carried both *PIK3CA* and *TP53* mutations (Additional file 1: Figure S1B). Specifically, 49% (678/1389) of the breast cancer samples with *PIK3CA* mutation also carried a *TP53* mutation (Additional file 1: Figure S1C). This suggested that the two genes may function cooperatively in breast cancers.

### *Pik3ca*H1047R;*Trp53*R270H;*MMTV-Cre* mice develop malignant mammary tumors with a shorter latency

To understand the cooperativity between *Pik3ca*H1047R and *Trp53*, we generated double mutant mice *Pik3ca*H1047R;*Trp53*R270H;*MMTV-Cre* by crossing *Pik3ca*H1047R;*MMTV-Cre* with *Trp53* R270H flox mice (Fig. 1a). *Trp53*R270H mutation in mouse is equivalent to the human *TP53*R273H dominant-negative DNA-binding domain hotspot mutation. We validated the expression of the *Pik3ca*H1047R and *Trp53*R270H in *Pik3ca*H1047R;*Trp53*R270H;*MMTV-Cre* mammary tumors by RNA-seq (Additional file 1: Figure S2). The *Pik3ca*H1047R;*MMTV-Cre* mice and the *Trp53* R270H;*MMTV-Cre* mice had median tumor-free survival time of 62 weeks and 52.9 weeks, respectively. However, the double mutant mice *Pik3ca*H1047R;*Trp53*R270H;*MMTV-Cre* showed a much shorter latency with the median tumor-free survival time of 36.6 weeks (*P* < 0.0001; Fig. 1b). Consistent with this, in a limited set of animals, we observed the time to tumor survival from the initial detection was rapid in the *Pik3ca*H1047R;*Trp53*R270H;*MMTV-Cre* double mutant mice compared to the single mutant mice (Additional file 1: Figure S3).

**Fig. 2:**
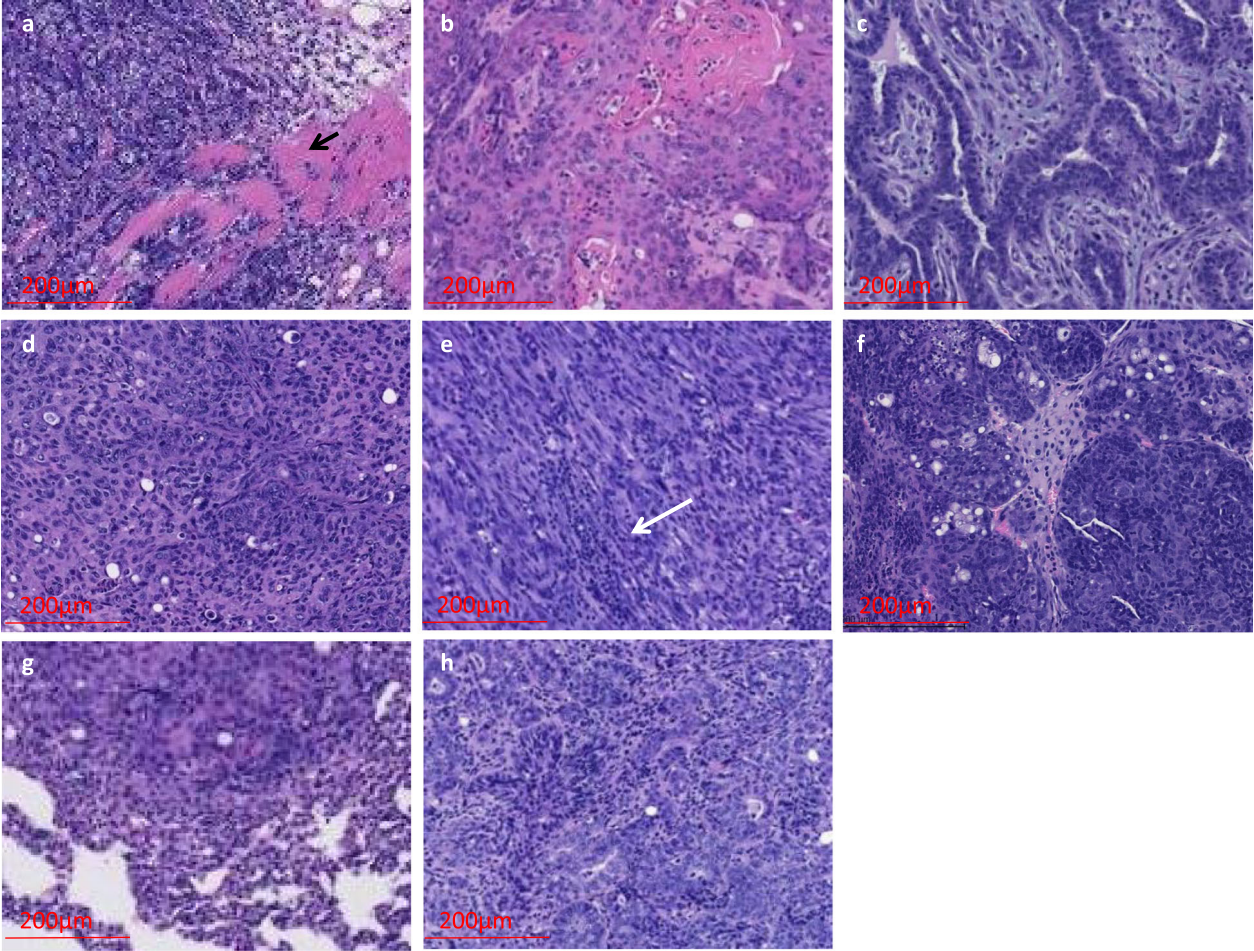
Histology of *Pik3ca*H1047R;*Trp53*R270H;*MMTV-Cre* primary mammary tumors and metastasis. **a** Invasive poorly differentiated adenocarcinoma (arrow indicates muscle fibers), **b** adenosquamous carcinoma, **c** fibroadenoma, **d** sarcomatoid adenocarcinoma, **e** sarcomatoid (spindle cell) carcinoma with lymphocyte infiltration (white arrow indicates site of lymphocyte infiltration), **f** poorly differentiated carcinoma, **g** metastatic lung carcinoma (from the same animal for which primary tumor is shown in panel **d**), and **h** metastatic lymph node (from the same animal for which primary tumor is shown in panel **e**)

The *Pik3ca*H1047R;*MMTV-Cre* animals generally develop benign fibroadenomas (77%) [7]. In contrast, we found the *Pik3ca*H1047R;*Trp53*R270H;*MMTV-Cre* double mutant mice developed primarily mammary adenocarcinomas (41%; Figs. 1c and 2). Additionally, they also developed moderate to poorly differentiated carcinomas (26%) and sarcomatoid (spindle cell) carcinomas (21%; Figs. 1c and 2). Histological analysis revealed that 15% (5/34) of the double mutant tumors had invasive features (Fig. 2a and Additional file 1: Figure S4A). Also, some tumors showed lymphocyte infiltration (Fig. 2e). The *Trp53*R270H;*MMTV-Cre* model also developed similar tumor types with the exception that no fibroadenomas were observed (Fig. 1c and Additional file 1: Figure S5). Among *Pik3ca*H1047R;*Trp53*R270H;*MMTV-Cre* animals, we found an animal that developed sarcomatoid adenocarcinoma with lung metastasis (Fig. 2g and Additional file 1: Figure S4G). We examined *Pik3ca*H1047R;*MMTV-Cre* mice (*n* = 4), *Pik3ca*H1047R; *Trp53*R270H;*MMTV-Cre* mice (*n* = 9), and *Trp53*R270H;*MMTV-Cre* mice (*n* = 2) for lymph node metastasis. We found evidence for lymph node metastasis in one *Pik3ca*H1047R;*Trp53*R270H;*MMTV-Cre* animal with sarcomatoid (spindle cell) carcinoma (Fig. 2h and Additional file 1: Figure S4H). Overall, ~ 21% (7/34) of the double mutant mice showed tumors with invasive or metastatic features. Also, one *Trp53*R270H animal with poorly differentiated carcinoma had metastasis in eight out of nine lymph nodes (Additional file 1: Figure S5F), suggesting that the *Trp53* mutation likely contributes to the development of metastasis.

To understand the different cell types in tumors, we tested the expression of basal (Krt5), luminal (Krt18), and mesenchymal (Vimentin) markers using immunofluorescence. We observed both luminal and basal cells and also mesenchymal cells in the tumors (Additional file 1: Figure S6). We also tested the tumors for progesterone receptor (Pgr) and estrogen receptor (Esr) positivity. One *Pik3ca*H1047R;*Trp53*R270H;MMTV-Cre poorly differentiated carcinoma showed a few Pgr-positive cells and was Esr negative, and another *Pik3ca*H1047R;*Trp53*R270H;MMTV-Cre sarcomatoid adenocarcinoma showed only a few Esr-positive cells (Additional file 1: Figure S6C and 6E). A *Trp53R*270H;MMTV-Cre sarcomatoid carcinoma (Additional file 1: Figure S6F) was Esr negative (data not shown).

### Single-cell RNA-seq identifies distinct cell types in *Pik3ca*H1047R;*Trp53*R270H;*MMTV-Cre* mammary tumors

**Fig. 3:**
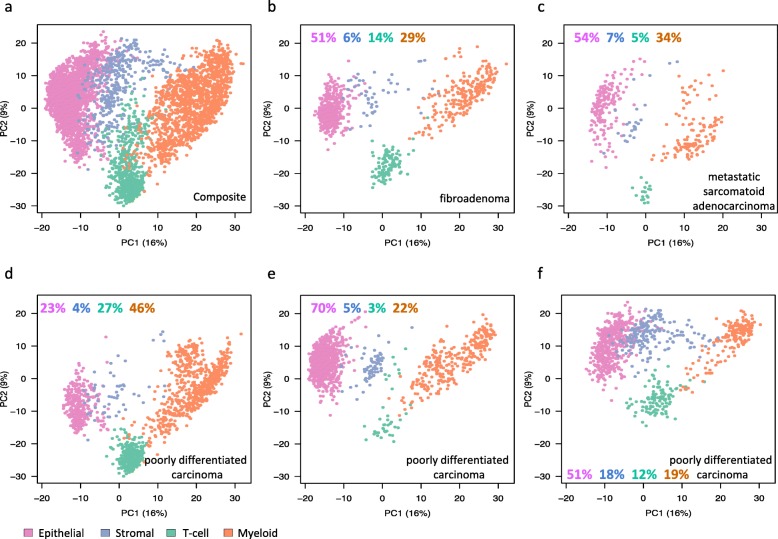
Single-cell RNA-seq analysis identified multiple cell types in mammary tumors. **a** PCA plot showing clustering of cells for five tumors analyzed as indicated. **b**–**f** The PCA clustering of the five individual tumors. **b** A fibroadenoma. **c** A metastatic sarcomatoid adenocarcinoma. **d**–**f** Three poorly differentiated carcinomas. The percentage of each cell types are shown in color-coded numbers

In order to further understand the cell types in tumors, we performed single-cell RNA-seq analysis on five tumor samples, including one *Pik3ca*H1047R;*MMTV-Cre* tumor and four *Pik3ca*H1047R;*Trp53*R270H;*MMTV-Cre* tumors. Principal component analysis (PCA) of single-cell RNA-seq data from 4995 total cells identified four distinct cell clusters in the tumors. They each predominantly expressed genes that are either epithelial, stromal, myeloid (monocytes and macrophages), or T cell types (Fig. 3a). The PCA profiles of the five individual tumors that included a benign fibroadenoma (*Pik3ca*H1047R;*Trp53*R270H;*MMTV-Cre*; Figs. 3b and 2c), a metastatic sarcomatoid adenocarcinoma (*Pik3ca*H1047R;*Trp53*R270H;*MMTV-Cre*; Figs. 3c and 2d), a malignant poorly differentiated carcinoma (*Pik3ca*H1047R;*Trp53*R270H;*MMTV-Cre*; Figs. 3d and 2f), and two other poorly differentiated carcinomas (*Pik3ca*H1047R;*Trp53*R270H;*MMTV-Cre*; Fig. 3e and *Pik3ca*H1047R;*MMTV-Cre* Fig. 3f) showed different proportions of cell types (Fig. 3b–f). Epithelial–mesenchymal transition (EMT) plays a central role in the multistep cascade leading to metastasis [18]. We assessed single-cell RNA-seq data for EMT-positive cells using an EMT score that is based on known EMT genes (*Snai1*, *Twist1*, *Zeb1*, and *Zeb2*; Additional file 1: Figure S7B). We found more cells undergoing EMT in the two malignant tumors compared to the fibroadenoma (37–38% vs 11%; Additional file 1: Figure S7).

### Identification of metastasis genes in *Pik3ca*H1047R;*Trp53*R270H;*MMTV-Cre* mammary tumors

**Fig. 4:**
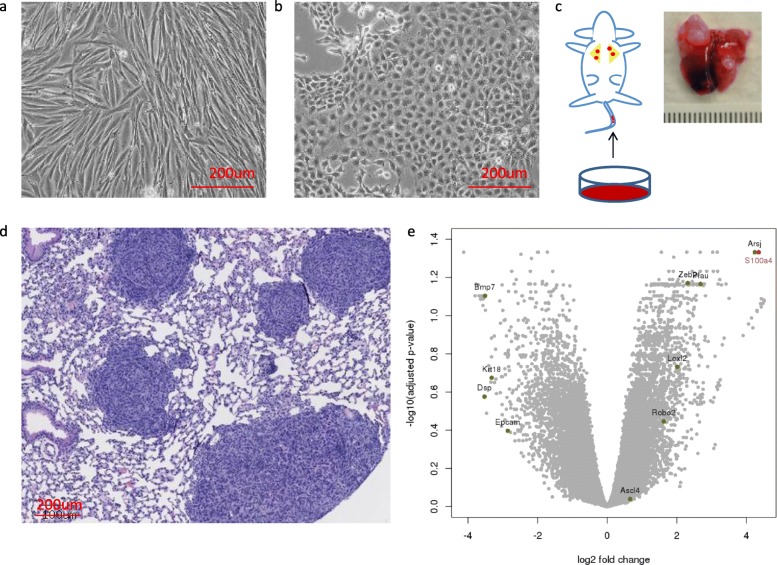
Differential expression analysis identified metastasis-associated genes. **a**, **b** Tumor-derived cell lines with different **a** spindle- or **b** cobblestone-like epithelial morphology. **c** Schematic of the experimental metastasis assay. Lung with metastatic tumor nodules shown. **d** Histology of the lung with tumor nodules. **e** Volcano plot depicting the differentially expressed genes when comparing metastatic sarcomatoid carcinomas vs adenocarcinomas (see the “Methods” section)

In order to understand the lung/lymph node metastatic phenotype observed in the double mutant *Pik3ca*H1047R;*Trp53*R270H;*MMTV*-*Cre* tumors, we established cell lines from primary sarcomatoid (spindle type) carcinomas. We also established cell lines from adenocarcinomas. The cells derived from the sarcomatoid tumors showed spindle morphology (Fig. 4a), while the cells derived from adenocarcinomas were cobblestone-like (Fig. 4b). We tested the metastatic potential of these cell lines using tail vein injection in mice and found that while the adenocarcinoma-derived cell lines did not metastasize, several spindle-like cell lines metastasized to the lung (Fig. 4c, d). While mice injected with three different adenocarcenoma cell lines (3 mice per line) all had normal lungs at 3 months post-injection, 50% of the mice that received three different sarcomatoid carcinoma cell lines (3 mice per line) developed lung nodules within 55 days and all had developed nodules by 74 days. An average of 3 visible nodules per 1 mm^2^ was present as assessed by histological analysis.

To understand the metastatic sarcomatoid carcinoma and non-metastatic adenocarcinomas, we analyzed their gene expression (Fig. 4e). We found epithelial marker genes, *Krt18*, *Epcam*, *Dsp*, were downregulated in the metastatic tumors, while genes that promote EMT, *Zeb2*, *Loxl2*, and *Plau* were upregulated. Interestingly, *BMP7*, an antagonist to EMT, which inhibits breast cancer metastasis [19], was a top gene that was downregulated in metastatic tumors. Also, pro-tumorigenic macrophage marker gene CD206 (*Mrc1*) and myeloid marker gene CD11b (*Itgam*) on average was 2.8× and 2.4× higher, respectively, in the metastatic tumors compared to non-metastatic carcinomas. Further, *S100a4* and *Arsj* were among the top overexpressed genes in the metastatic tumors. *Arsj* is a sulfatase and its precise role in metastasis requires further investigation.

In this study, we further investigate the role of *S100a4*, a gene previously reported to be differentially expressed in highly metastatic mouse mammary carcinomas [20]. In humans, *S100A4* is expressed during embryonic development in mesenchymal tissues, fetal macrophages, and trophoblasts, while in the adult stage, the expression is mainly restricted to immune cells such as macrophages, neutrophils, certain types of lymphocytes, and endothelial cells [21]. Analysis of *S100A4* expression in human breast cancers showed that its levels were higher in tumors carrying *PIK3CA* H1047R or *TP53* R270H or both compared to tumors without these mutations (Additional file 1: Figure S8). Previous studies have reported overexpression and association of *S100A4* with tumor aggressiveness, metastasis, and poor survival in patients in multiple cancers [22, 23].

We assessed the expression of *S100a4* in our single-cell RNA-seq data and found that all the three tumors and their constituent cell groups, epithelial, stromal, myeloid, and T cells, expressed *S100a4* (Additional file 1: Figure S7C). Moreover, we found more *S100a4* expressing cells in the two malignant tumors compared to fibroadenoma (51–56% vs 26%; Additional file 1: Figure S7C).

### CRISPR/Cas9 knockout *S100a4* gene adversely affects metastatic potential

**Fig. 5:**
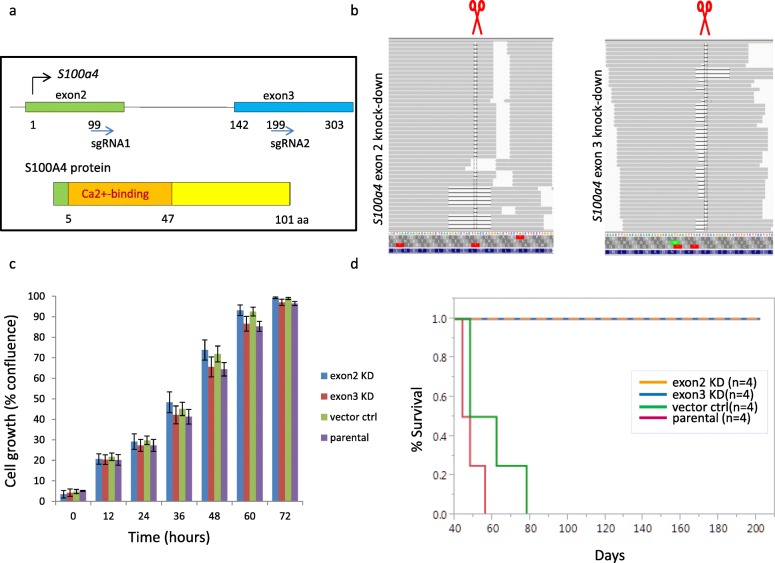
*S100a4* knockout impairs metastasis. **a** Gene and protein structure of *S100a4* in the mouse. **b** RNA-seq data confirming *S100a4* knockout. **c** Proliferation of the knockout lines. **d** Kaplan–Meier plot depicting survival of experimental metastatic assay. Cell line established from the primary tumor in the *Pik3ca*H1047R*;Trp53*R270H*;MMTV-Cre* double mutant mice with lung metastasis was used to generate the knockout lines

The human S100A4 gene composed of four exons, with exons 3 and 4 encompassing the protein coding sequence, is located on chromosomal region 1q21. In mouse, *S100a4* spans three exons where the first exon encodes the 5′ (untranslated region) UTR. *S100a4* encodes a 101 amino acid protein that belongs to the S100 Ca^2+^ binding family (Fig. 5a). We knocked out *S100a4* using two lentiviral constructs expressing *S100a4* sgRNA1, corresponding to exon 2, and sgRNA2, corresponding to exon 3 (Fig. 5a). A cell line established from the primary tumor in the *Pik3ca*H1047R*;Trp53*R270H*;MMTV-Cre* double mutant mice with lung metastasis was used to generate the knockout lines. Using RNA-seq, we found evidence for the presence of protein truncating mutation in *S100a4* (Fig. 5b) in 93.2 to 99.9%, of the transcripts (Additional file 1: Table S1). Also, we confirmed the S100a4 loss by Western blot analysis (Additional file 1: Figure S9). We assessed the effect of the *S100a4* knockout on cell proliferation by live cell imaging and found that it did not affect cellular proliferation when compared to the parental wildtype control line (Fig. 5c).

We analyzed the gene expression profile of the *S100a4* knockout lines and found many gene expression changes compared to the parental line and vector control line using RNA-seq (Additional file 1: Figure S10). The epithelial marker gene *Epcam* was upregulated in the knockout lines compared to the parental line. In contrast, *Sox4*, the master regulator of EMT, and additional EMT genes such as *Met*, *Zeb2*, *Etv1*, and *Idh1* were downregulated. Also, several matrix metalloproteinase genes, *Mmp3*, *Mmp9*, and *Mmp16*; extracellular matrix genes, *Col3a1*, *Lamc2*, *Loxl2*, and *Bgn*; and genes for adhesion molecules, *Cdh13*, *Itgb1*, and *Itgb8*, were downregulated. Interestingly, a long non-coding RNA gene *Zfas1* was significantly upregulated in our *S100a4* knockout lines. Previously, *ZFAS1* was shown to be downregulated in human breast cancer cells and *ZFAS1* overexpression led to reduced cell migration and invasion through inhibition of EMT [24]. Two chemokine genes, *Ccl2* and *Ccl7*, which function in recruiting immune cells, were downregulated. In the knockout lines, several other pathways were affected, including P53 pathway (*Mdm4*, *Crebbp*, *Kat2b*), apoptosis pathway (*Bcl2l1*, *Birc3*, *Cflar*, *Xiap*, *Traf1*), Ras/Pi3k pathway (*Braf*, *Jun*, *Pik3ca*, *Pik3r3*, *Pten*, *Sos2*), Tgfβ pathway (*Tgfb2*, *Tgfbr1*, *Smad5*), Wnt pathway (*Fzd9*, *Ctnnal1*, *Prkca*, *Wisp1*), and hypoxia pathway (*Hif1a*). *Myosin 1b*, which regulates the actin crosslinking, is downregulated too. Interestingly, some genes that we previously found upregulated in the metastatic tumors, e.g., *Arsj* (Fig. 4), were also downregulated in the knockout lines (Additional file 1: Figure S10).

We tested the metastatic potential of the parental and knockout tumor cell lines using a syngeneic C57BL/6 tail vein injection model. We found that the parental tumor cell line developed from a *Pik3ca*H1047R;*Trp53*R270H;*MMTV-Cre* metastatic tumor (Fig. 2d) when injected into mouse tail vein showed high metastatic potential (Fig. 4c, d). We found that compared to the parental line, mice receiving the *S100a4* knockout lines did not develop lung nodules even until day 200 as assessed by necropsy (Fig. 5d). The mice receiving the parental or vector control line showed a marked reduction in survival (*n* = 4) and had developed lung nodules consistent with the expected metastatic potential of these lines.

## Discussion

Genetically engineered mice (GEM) are powerful tools for understanding cancer development. In a set of 4485 breast cancer samples, we found *PIK3CA* (31%) and *TP53* (62%) to be the top two mutated genes [13]. In this study, we assessed the consequence of breast cancer development in a mouse expressing *Pik3ca*H1047R and a dominant-negative *Trp53* mutant R270H. We found that the two genes cooperate in breast cancer development by drastically reducing the latency for tumor development (Fig. 1b). This is consistent with a previous study where transgenic *Pik3ca* mice, *R26-Pik3ca*H1047R when crossed to a *Trp53* null mouse, led to decreased survival [5].

Histological analysis identified multiple breast tumor types including adenocarcinoma, adenosquamous carcinoma, moderately to poorly differentiated carcinoma, and sarcomatoid (spindle cell) carcinoma. About 21% of the tumors were found to be sarcomatoid carcinoma. Similar diverse tumor histological tumor types were reported in a previous study [5]. In humans, metaplastic sarcomatoid carcinomas are mostly triple receptor-negative breast cancers (TNBC) and appear more aggressive than other TNBCs [25]. Metaplastic sarcomatoid carcinomas comprise a small but histologically diverse group of invasive breast cancers that share an unusual morphologic characteristic in that a part or all of the tumor cells appear to have undergone transformation to a non-glandular epithelial or mesenchymal cell type [26]. Also, there were increases in *PIK3CA* mutations, *AKT* copy numbers, and phosphorylation of PI3K/AKT pathway components in metaplastic carcinomas compared to most other breast tumor subtypes [26]. Further, in our model, we found metastasis in a few animals with primary tumors displaying sarcomatoid features. Several cell lines derived from sarcomatoid tumors showed spindle cell morphology and were found to be metastatic. *MMTV-PyMT*, *MMTV-Neu* (*Her2*), *MMTV-PyMT*;*Akt1*−/−, *MMTV-PyMT*;*CD44*−/−*MMTV-Cre*; *Trp53*fl/fl, and *MMTV-Cre*; *Brca*1fl/fl; *Trp53*+/− mice were previously shown to develop lymph node and lung metastases [27, 28]. Additionally, *WAP-Cre;Pten* fl/fl; *Trp53*+/R270H mutant tumor cells were reported to show lung metastases [29]. Our model provides an additional system to study metastasis and develop therapeutic strategies for *PIK3CA*/*TP53* double-positive cancers.

Single-cell RNA-seq analysis of the tumors identified epithelial, stromal, myeloid, and T cell types in the tumor. Metastatic tumors contained a much higher percentage of EMT-signature positive and *S100a4*-expressing cells among the epithelial, stromal, and myeloid clusters. Using CRISPR/CAS9-mediated knockout of *S100a4*, we demonstrate its relevance in metastasis. Genomic alterations at chromosomal region 1q21, in which most *S100* genes are clustered, are frequently observed in human epithelial tumors [21]. *S100A4* is overexpressed in multiple cancers such as breast, lung, colorectal, esophageal, gastric, pancreatic, hepatocellular, gallbladder, and bladder cancers. Increased expression of *S100A4* is associated with aggressiveness of a tumor, metastasis, and poor patient survival [22, 23]. Our analysis of *S100A4* expression in human breast cancers showed that its levels were higher in tumors carrying *PIK3CA*H1047R or *TP53*R270H or both compared to tumors without these mutations (Additional file 1: Figure S8) though they were not statistically different. Given that the number of double mutant tumors with *S100A4* expression data in this set (Additional file 1: Figure S8) is much lower than the single mutant tumors, analysis of a larger cohort of double mutant tumors with the tumor stage-specific information will help further confirm the role of *S100A4* in metastasis. Overall, targeting *S100A4* alone or in combination with driver mutations such as *PIK3CA* may provide a durable therapeutic intervention opportunity in aggressive breast cancers.

## Conclusions

*Pik3ca*H1047R;*Trp53*R270H;*MMTV-Cre* mouse described herein provides a preclinical model to mimic a subtype of human breast cancers and to investigate the genetic cooperation that drives primary mammary tumorigenesis and metastasis. S100a4, the critical molecule driving metastasis in this model, has the potential in diagnostics and therapeutic targeting.

## Supplementary information

**Additional file 1: Figure S1.** Frequency of *TP53* and *PIK3CA* mutations in human breast cancer samples. A. *TP53* and *PIK3CA* are the two most frequently altered genes in breast cancers. B. Frequency of *PIK3CA* and *TP53* double mutant breast cancer samples. C. Frequency of *TP53* mutations in breast cancer samples with *PIK3CA* mutations. **Figure S2.** Expression of *Pik3ca*H1047R (A) and *Trp53*R270H (B) alleles in a *Pik3ca*H1047R/+;*Trp53*R270H/+;*MMTV-Cre* tumor as assessed by RNA-seq. **Figure S3.** Tumor progression in *Pik3ca*H1047R*;Trp53*R270H*;MMTV-Cre* mice, *Pik3ca*H1047R*;MMTV-Cre* mice and *Trp53*R270H*;MMTV-Cre* mice. **Figure S4.** Lower magnification of the panels in Fig. 2 in the same order. **Figure S5.** Histology of *Trp53*R270H;*MMTV-Cre* primary mammary tumors. A. adenocarcinoma, B. adenosquamous carcinoma, C. sarcomatoid (spindle cell) carcinoma, D. invasive poorly differentiated carcinoma on bone (arrow indicates bone tissue), E. poorly differentiated carcinoma, and F. metastatic lymph node (from the same animal bearing primary tumor in panel E). **Figure S6.** Immunofluorescence staining of a poorly differentiated *Pik3ca*H1047R*;Trp53*R270H*;MMTV-Cre* carcinoma with focal sarcomatoid features (A-C) or sarcomatoid *Pik3ca*H1047R*;Trp53*R270H*;MMTV-Cre* (D-E) adenocarcinoma or sarcomatoid *Trp53*R270H*;MMTV-Cre* carcinoma (F). Staining for Krt18 (green), Krt5 (red), Vimentin (green), progesterone receptor (Pgr, green) and estrogen receptor(Esr, red) are shown. DAPI is shown in blue. **Figure S7.** Cellular heterogeneity of *Pik3ca*H1047R;*Trp53*R270H;*MMTV-Cre* tumors. A. Same as Fig. 3B-D. EMT signature positive (B) and *S100a4* positive (C) for cells in panel A. The numbers indicate the percentages of cell types (A), percentage of EMT positive cells (B) and *S100a4* positive cells (C). **Figure S8.** Expression level of *S100a4* in breast cancer TCGA data set (www.cbioportal.org). RSEM*: RNA-Seq by Expectation Maximization (BMC Bioinformatics 2011, 12:323). **Figure S9.** S100a4 protein levels in CRISPR/Cas9 knockout lines assessed by Western blotting. **Figure S10.** Log2 RPKM heatmap comparing RNA-seq based expression of select genes in the *S100a4* knockout, parental and vector control cell lines. **Table S1.** Summary of allelic frequency, in the *S100a4* knockout lines.

## Data Availability

Materials as appropriate will be made available.

## Abbreviations

MMTV: Mouse mammary tumor virus
EMT: Epithelial–mesenchymal transition
CGP: Comprehensive genomic profiling
CLIA: Clinical Laboratory Improvement Amendments
GA: Genomic alterations
IACUC: Institutional Animal Care and Use Committee
HBSS: Hanks Balanced Buffer Solution
UTR: Untranslated region
TNBC: Triple receptor-negative breast cancers
