# Supplementary material for: S100a4 upregulation in Pik3caH1047R;Trp53R270H;MMTV-Cre-driven mammary tumors promotes metastasis
Source: Breast Cancer Res. 2019 Dec 27;21:152. doi: 10.1186/s13058-019-1238-5 (PMC6935129; doi:10.1186/s13058-019-1238-5)
Supplement: Supplementary file 1 — Additional file 1: Figure S1. Frequency of TP53 and PIK3CA mutations in human breast cancer samples. A. TP53 and PIK3CA are the two most frequently altered genes in breast cancers. B. Frequency of PIK3CA and TP53 double mutant breast cancer samples. C. Frequency of TP53 mutations in breast cancer samples with PIK3CA mutations. Figure S2. Expression of Pik3caH1047R (A) and Trp53R270H (B) alleles in a Pik3caH1047R/+;Trp53R270H/+;MMTV-Cre tumor as assessed by RNA-seq. Figure S3. Tumor progression in Pik3caH1047R;Trp53R270H;MMTV-Cre mice, Pik3caH1047R;MMTV-Cre mice and Trp53R270H;MMTV-Cre mice. Figure S4. Lower magnification of the panels in Fig. 2 in the same order. Figure S5. Histology of Trp53R270H;MMTV-Cre primary mammary tumors. A. adenocarcinoma, B. adenosquamous carcinoma, C. sarcomatoid (spindle cell) carcinoma, D. invasive poorly differentiated carcinoma on bone (arrow indicates bone tissue), E. poorly differentiated carcinoma, and F. metastatic lymph node (from the same animal bearing primary tumor in panel E). Figure S6. Immunofluorescence staining of a poorly differentiated Pik3caH1047R;Trp53R270H;MMTV-Cre carcinoma with focal sarcomatoid features (A-C) or sarcomatoid Pik3caH1047R;Trp53R270H;MMTV-Cre (D-E) adenocarcinoma or sarcomatoid Trp53R270H;MMTV-Cre carcinoma (F). Staining for Krt18 (green), Krt5 (red), Vimentin (green), progesterone receptor (Pgr, green) and estrogen receptor(Esr, red) are shown. DAPI is shown in blue. Figure S7. Cellular heterogeneity of Pik3caH1047R;Trp53R270H;MMTV-Cre tumors. A. Same as Fig. 3B-D. EMT signature positive (B) and S100a4 positive (C) for cells in panel A. The numbers indicate the percentages of cell types (A), percentage of EMT positive cells (B) and S100a4 positive cells (C). Figure S8. Expression level of S100a4 in breast cancer TCGA data set (www.cbioportal.org). RSEM*: RNA-Seq by Expectation Maximization (BMC Bioinformatics 2011, 12:323). Figure S9. S100a4 protein levels in CRISPR/Cas9 knockout lines assessed by W [file 13058_2019_1238_MOESM1_ESM.pdf]

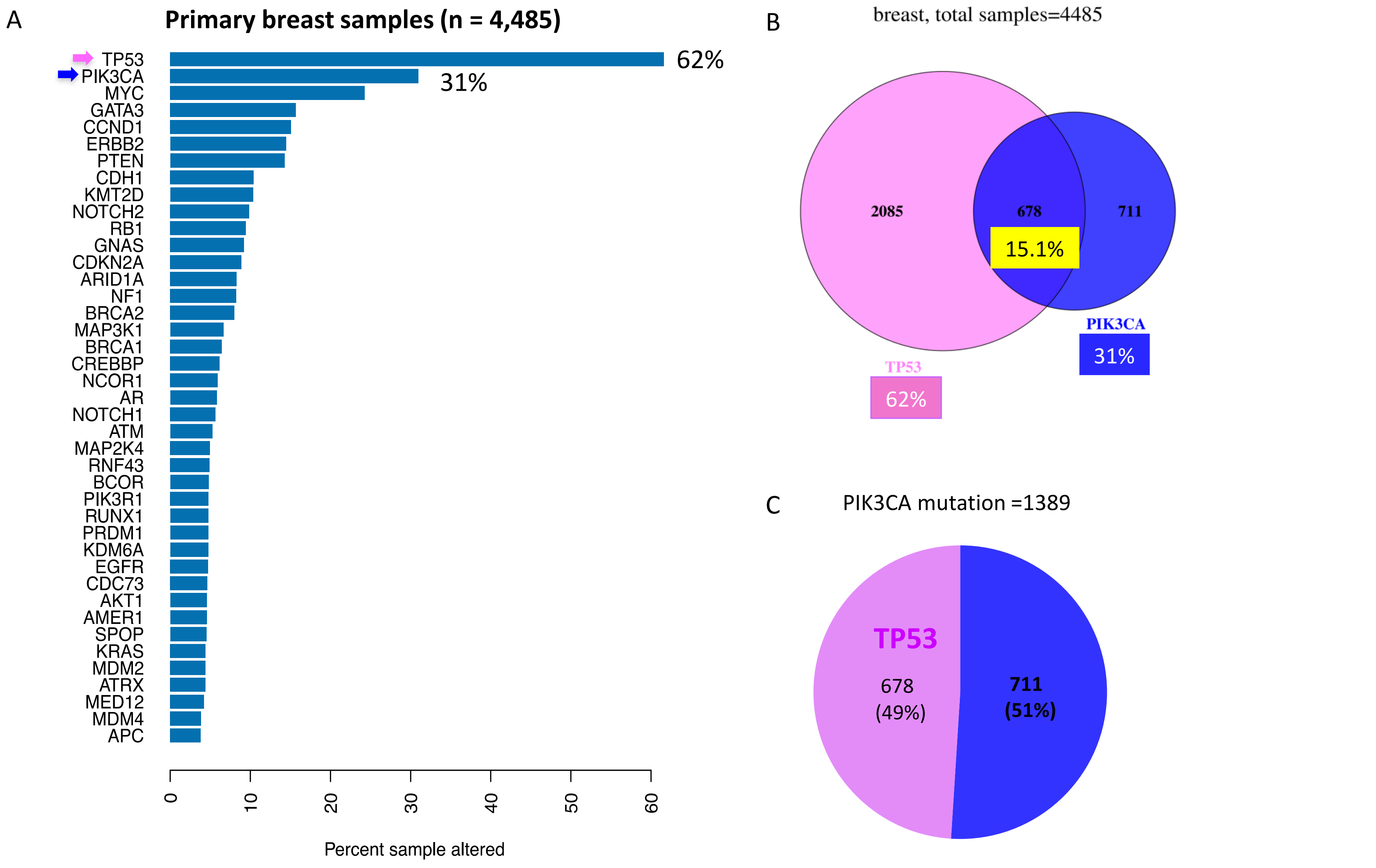

Supplementary Figure 1

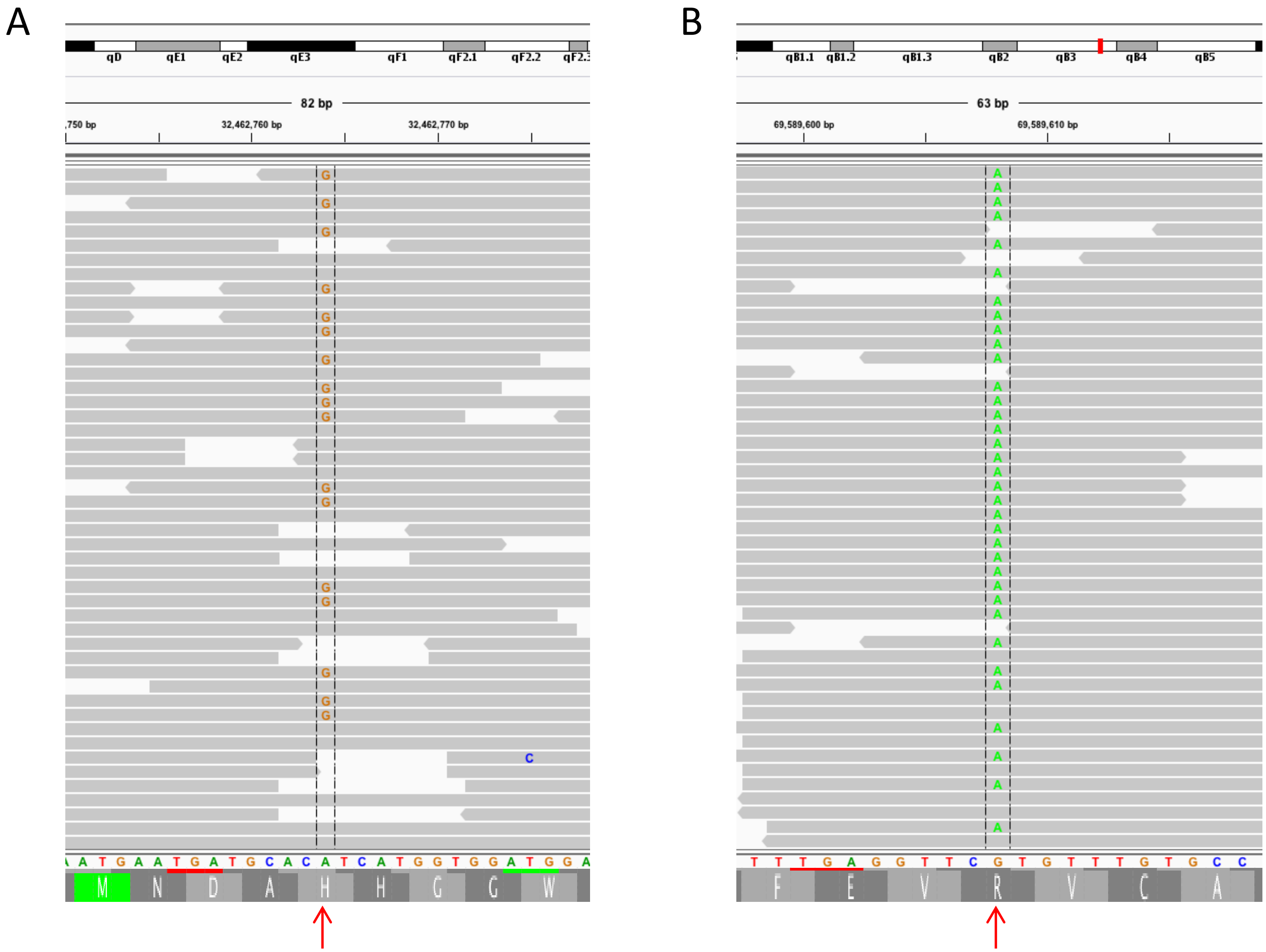

CAT to CGT for *Pik3ca*H1047R

CGT to CAT for *Trp53*R270H

Supplementary Figure 2

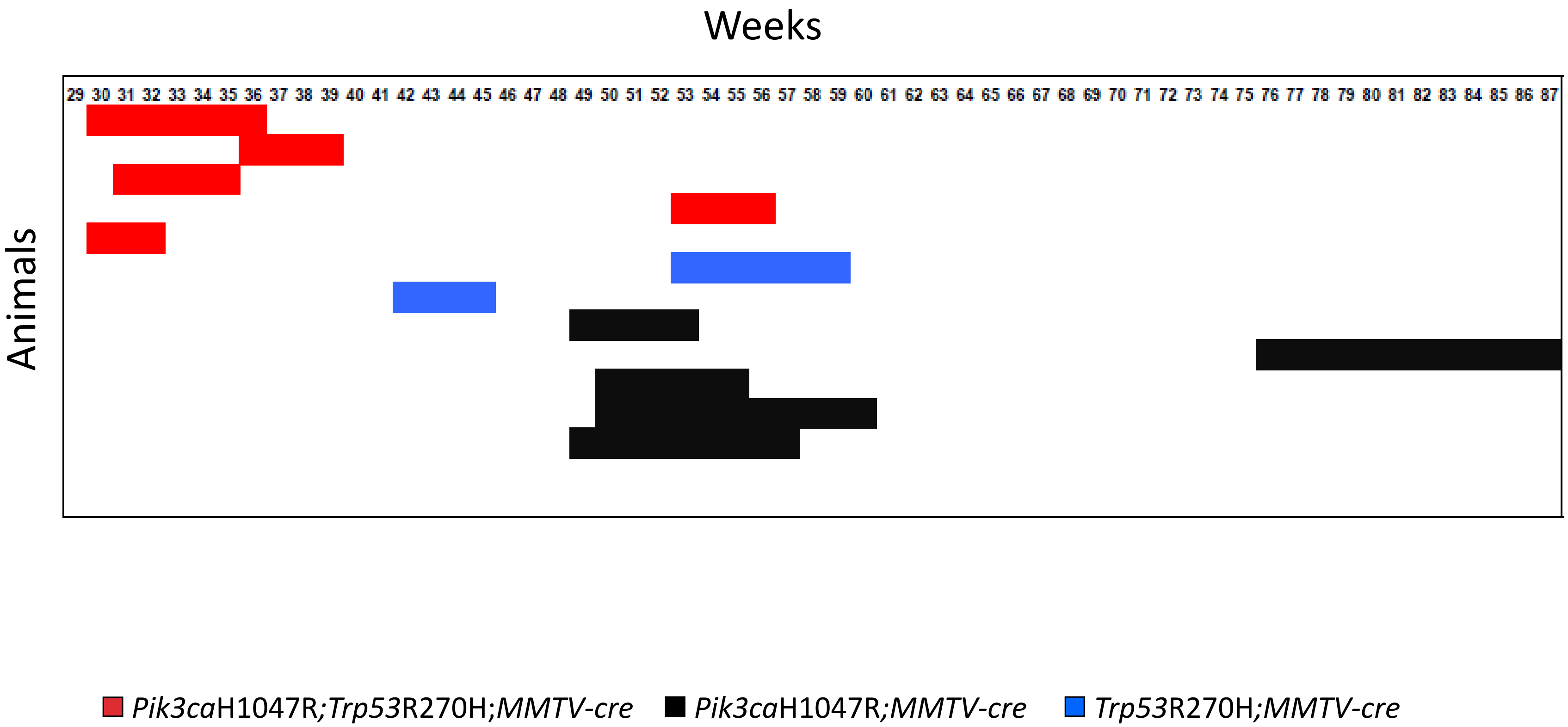

Supplementary Figure 3

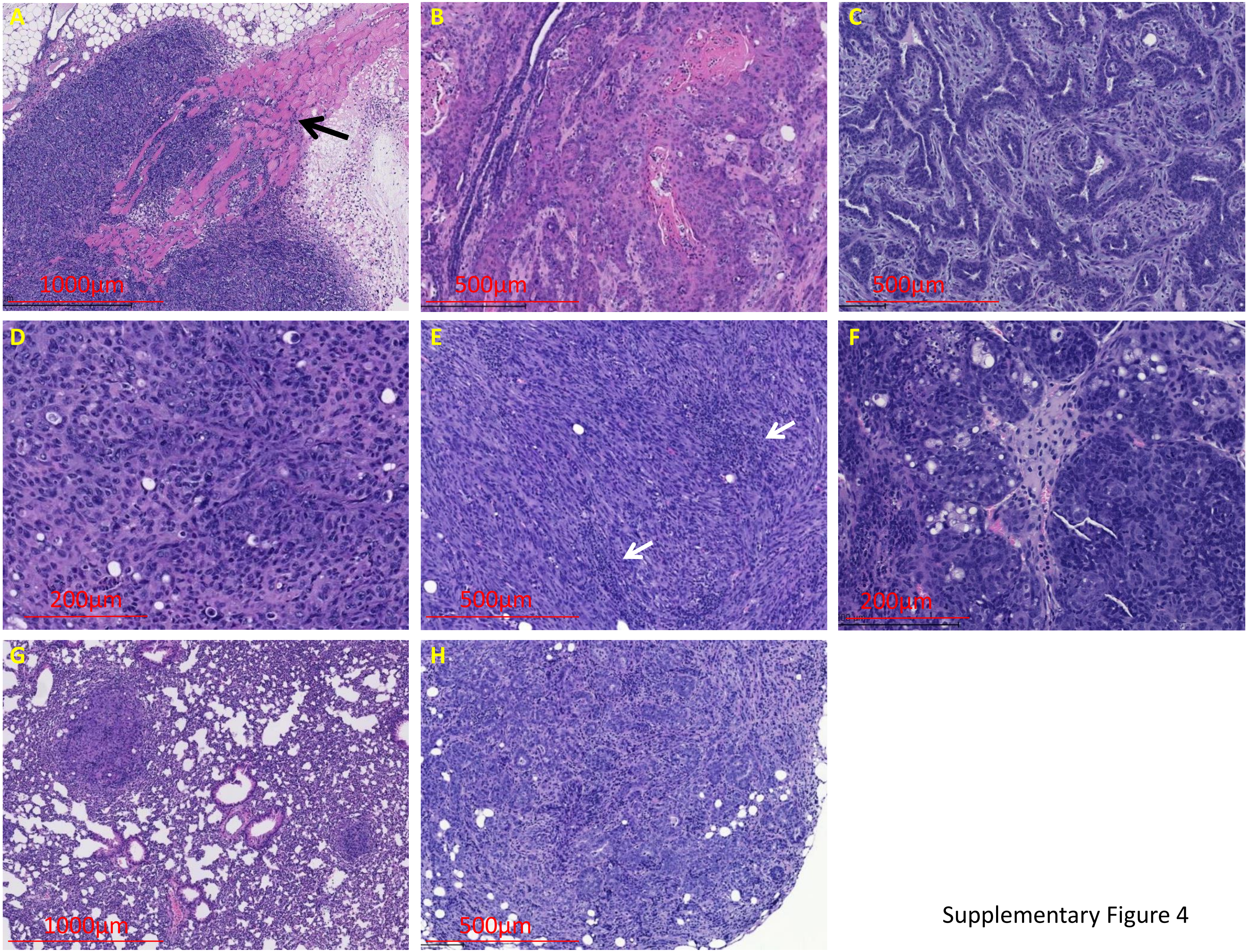

Supplementary Figure 4

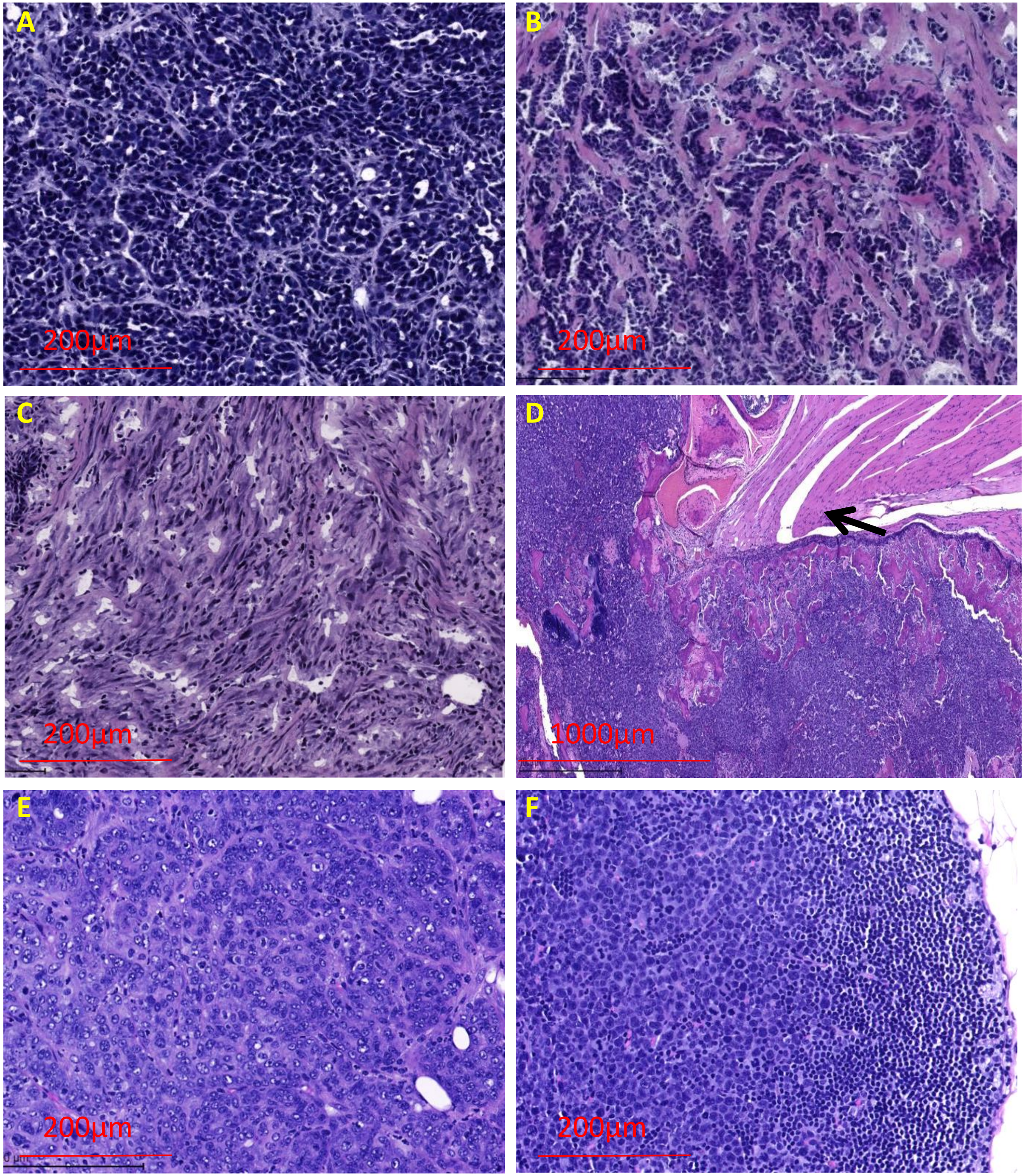

Supplementary Figure 5

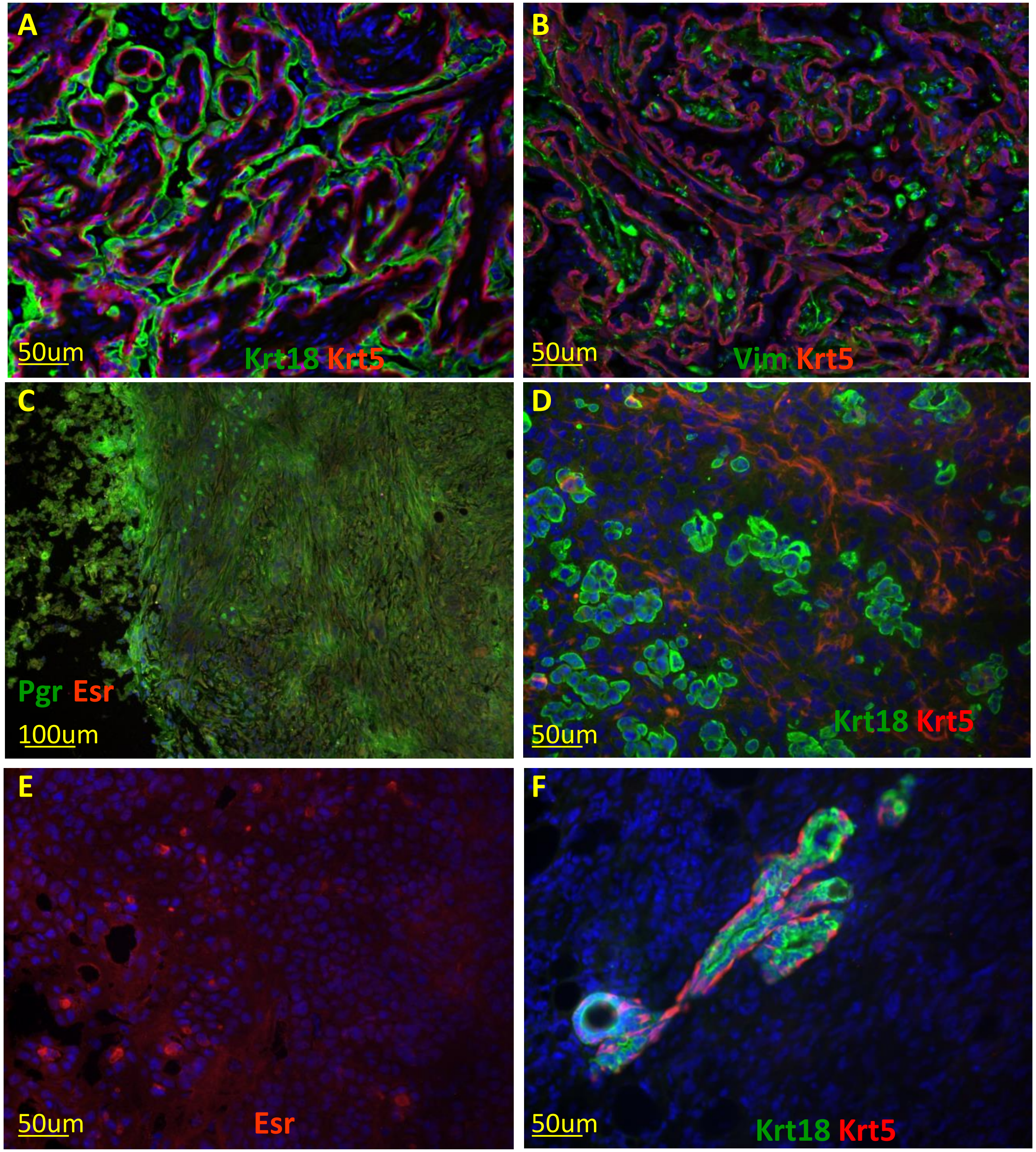

Supplementary Figure 6

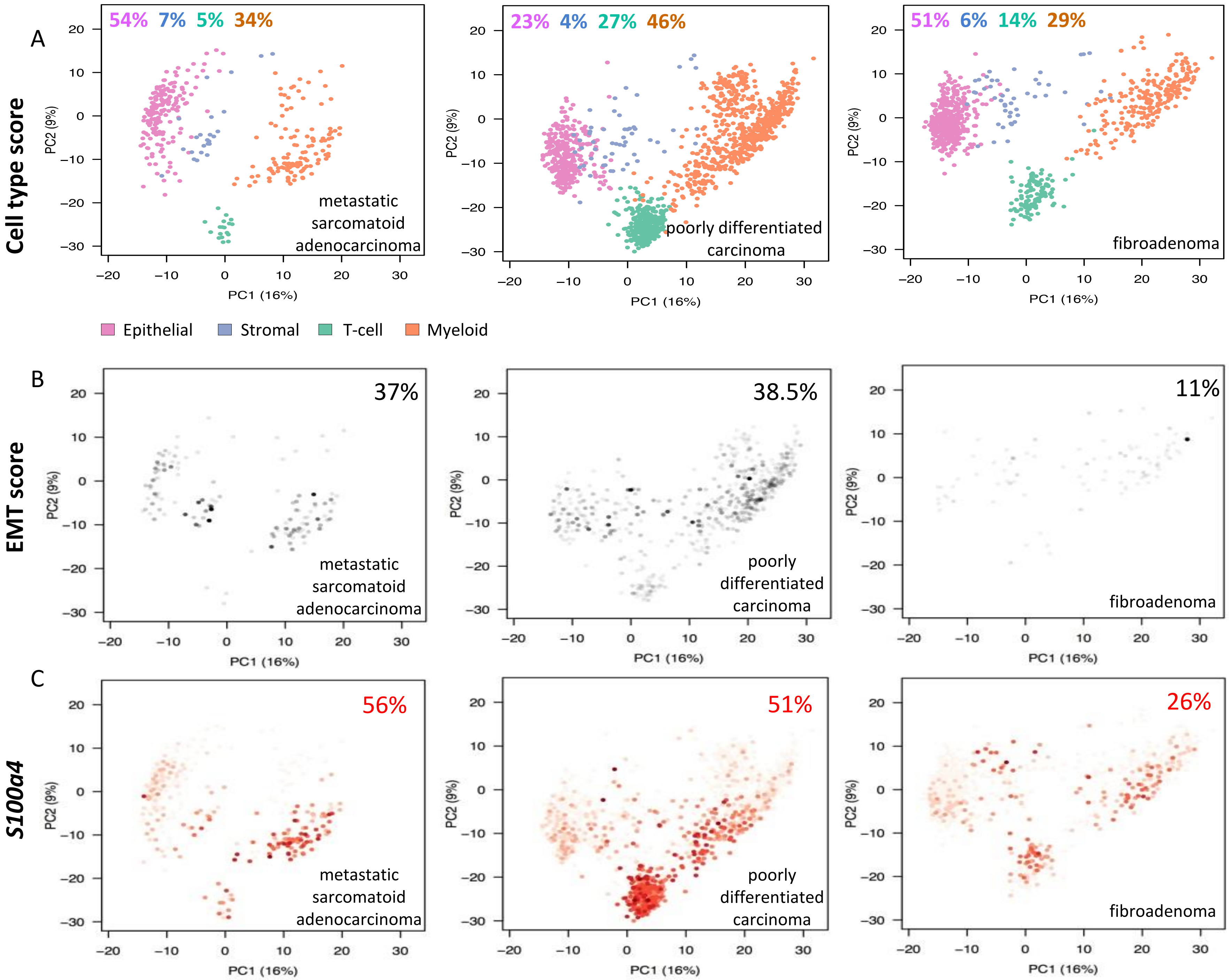

Supplementary Figure 7

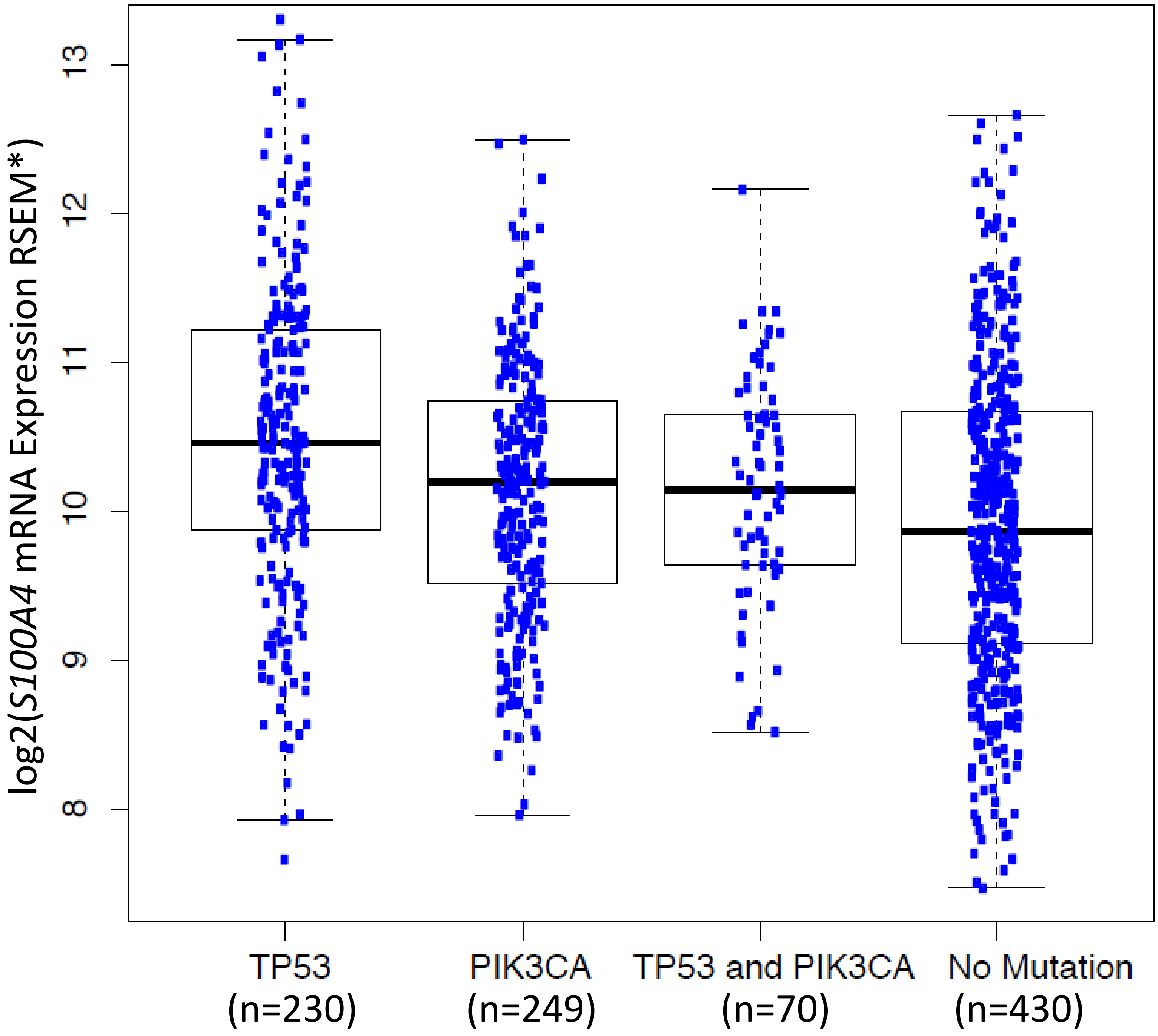

Supplementary Figure 8

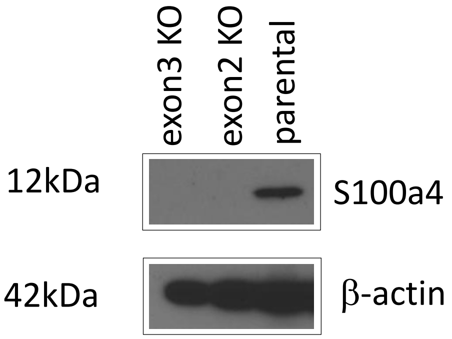

Supplementary Figure 9

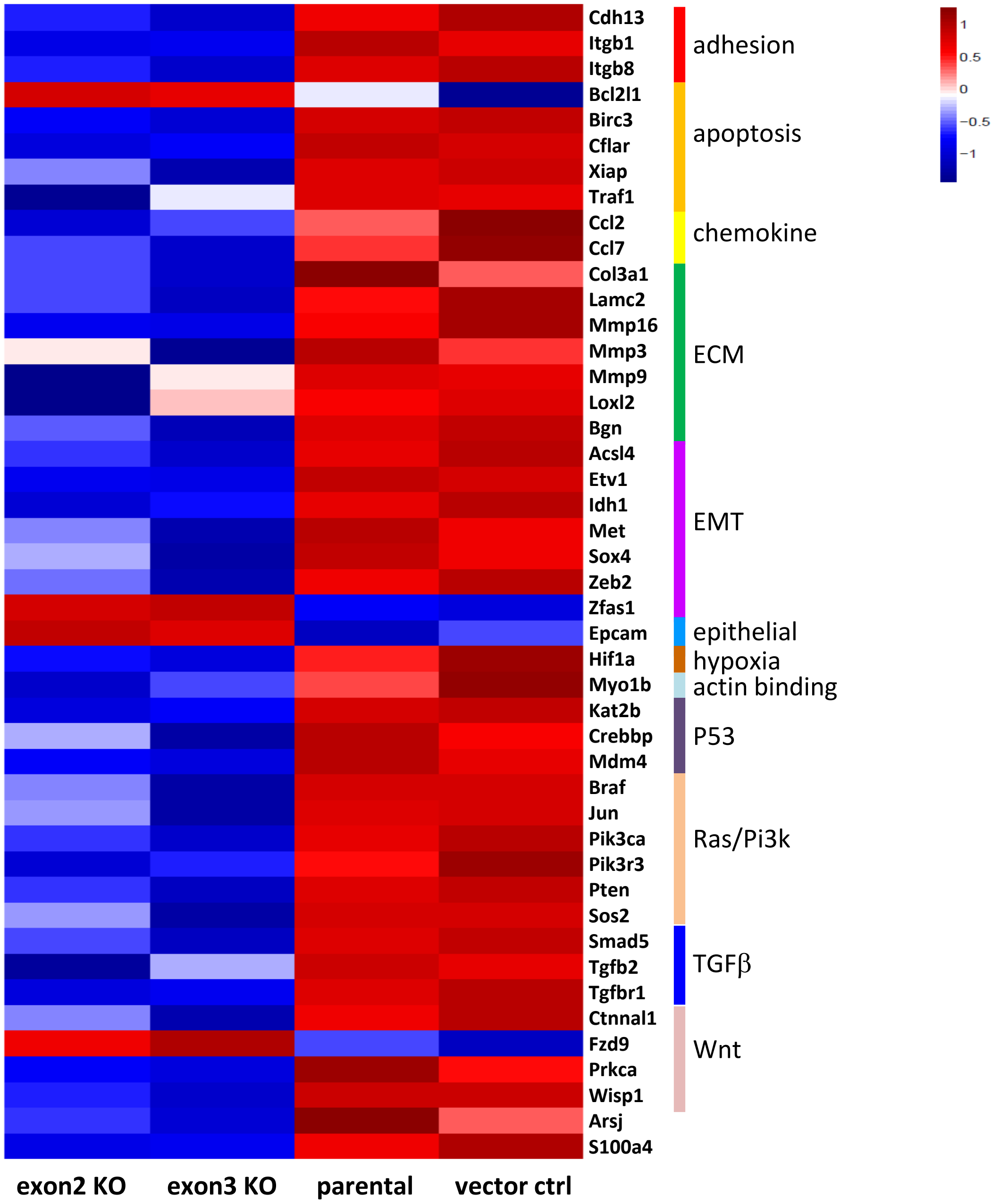

Supplementary Figure 10

Supplementary Table 1. Summary of allelic frequency in the *S100a4* knockout lines

	exon2 KO	exon3 KO	outcome
wt	6.8%	0.1%	
del 1bp	37.7%	49.4%	truncation of protein
del 2bp	3.1%	23.2%	truncation of protein
del 3bp	0.6%	8.9%	delete 1 amino acid
del 4bp		12.8%	truncation of protein
del 12bp		5.2%	delete 4 amino acids
del 15bp	51.9%		delete 5 amino acids
total deletion	93.2%	99.9%	
